# Screening and biological characteristics of biocontrol endophytes against *Idesia polycarpa* stem canker

**DOI:** 10.3389/fpls.2026.1860793

**Published:** 2026-07-15

**Authors:** Juan Wang, Yiting Song, Wenyan Zhi, Lisha Fang, Li Dai, Zhi Li, Xiaodong Geng, Qifei Cai, Yongyu Ren, Yaohui Liu, Zhen Liu, Yanmei Wang

**Affiliations:** 1 College of Forestry, Henan Agricultural University, Zhengzhou, China; 2 National Forestry and Grassland Administration Key Laboratory for Central Plains Forest Resources Cultivation, Zhengzhou, China; 3 College of Forestry, Xinyang Agriculture and Forestry University, Xinyang, China; 4 Henan Province Engineering Technology Research Center for Idesia, Zhengzhou, China

**Keywords:** *Aneurinibacillus migulanus*, biological control, *Botryosphaeria dothidea*, *Idesia polycarpa*, stem canker

## Abstract

Stem rot of *Idesia polycarpa* Maxim., caused by *Botryosphaeria dothidea*, induces cankers that lead to tree death, severely affecting tree growth and fruit yield, and constraining the development of the *I. polycarpa* industry. This study aimed to screen for endophytes antagonistic to the stem rot pathogen from healthy trees of the cultivar ‘Yuji’ *I. polycarpa* and to investigate their biological characteristics, in order to provide a basis for biological management of the disease. A total of 105 endophytic fungal strains and 74 bacterial strains were isolated and purified from healthy *I. polycarpa* trees. Following screening by the plate confrontation method, the isolates were analyzed and identified via ITS and 16S rRNA sequencing. The biological characteristics of the selected biocontrol strain were further investigated.The biocontrol endophytic strain B47 was obtained. It shared 97.58%–97.83% sequence similarity with known species of the genus *Aneurinibacillus* and was named *Aneurinibacillus* sp. B47. This strain exhibited an inhibition rate of 84.56% against *B. dothidea* and a relative control efficacy of 44.44% on two‑year‑old *I. polycarpa* plants. It could grow and reproduce normally at temperatures of 5–50°C, pH 3–10, and salinity below 7%. It was an aerobic, catalase‑positive bacterium with strong growth potential.Aneurinibacillus sp. B47 exhibited strong antagonistic activity, broad environmental adaptability, and rapid growth, indicating its potential for development as a biocontrol agent against stem rot of *Idesia polycarpa* Maxim.

## 1 Introduction

*Idesia polycarpa* is a deciduous oil tree of the genus *Idesia* in the family Flacourtiaceae (Li et al., 2019; Xiang et al., 2023). It is distributed in most parts of China and is an important biomass energy tree species (Wang et al., 2023; Niu et al., 2025). *I. polycarpa* has earned a reputation as oil depot on the tree due to its tolerance to barren conditions, strong drought and cold resistance, high fruit yield, and high oil content (Fang et al., 2020; Tian et al., 2020). Meanwhile, it’s a good raw material for use as an edible oil and industrial oil (Song et al., 2022). Due to its tall and upright tree shape, the fruit turns bright red or orange–yellow when mature, it has great ornamental value and is also a high-quality ornamental and environmentally beneficial tree species (Jiang et al., 2022; Li et al., 2022). As a woody oil tree species, *I. polycarpa* has great development prospects in multiple fields such as forestry, food, medicine and ecology. The National Food and Strategic Reserves Administration of China has officially formulated and implemented a number of national standards for *I. polycarpa* and *I. polycarpa* oil (National Technical Committee on Cereals and Oils Standardization, 2018a, b; Li et al., 2022). However, during the growth of *I. polycarpa*, it is often attacked by various diseases, such as stem canker disease, gray leaf spot disease and ring rot disease, etc., which affects its growth quality and fruit yield. Previous studies have found that *I. polycarpa* over five years old are more likely to be infected to death because of the stem canker disease caused by *Botryosphaeria dothidea* (Fang et al., 2024).

*Botryosphaeria* species are widely distributed, important tree canker and death-inducing pathogens worldwide (Ma et al., 2023; Li, 2024). *B. dothidea* is the most common *Botryosphaeria* fungus and is an important pathogen that causes canker and fruit rot in various fruit trees and forest trees (Zang et al., 2021; Wang, 2023). Blister canker caused by *B. dothidea* poses a severe threat to poplar trees (Liu et al., 2024), with infection and mortality rates exceeding 95% and 70%, respectively, resulting in significant damage to ecosystems (Ruess et al., 2021). Kiwifruit rot occurred widely in the major production regions of China, with an average incidence of 20%-50%, leading to substantial yield and economic losses. *Bacillus velezensis* RT-30 could be used as a biocontrol agent against kiwifruit rot (Yue et al., 2026). Aetiological studies have confirmed that the stem canker of *I. polycarpa* is a fungal disease which caused by *B. dothidea* (Fang et al., 2024). In recent years, the incidence of stem canker caused by *I. polycarpa* in plantation of Henan and Hubei Provinces in China has increased. The disease causes dead branches, discoloration, wood decay, and even tree death, which seriously affects the seedling production and fruit yield. This seriously restricts the development of the *I. polycarpa* industry. Currently, the management and suppression of stem rot rely mainly depend on physical and chemical control methods. Over time, however, the pathogen may develop resistance to fungicides and other chemical agents, and such methods can cause considerable environmental harm (Pan et al., 2022). It is therefore necessary to replace chemical agents with environmentally friendly approaches, such as antagonistic microorganisms, which are of great significance for controlling plant diseases and for practical application (De Simone et al., 2021; Dai et al., 2022).

Endophytes refer to bacteria or fungi that complete part or all of their life cycle in plants, achieve symbiotic relationships with them, coevolve, and do not harm the host plants (Ali et al., 2022; Mishra et al., 2022). They are ubiquitous in plants, and the community compositions of endophytes are also very different because of the different hosts and host environments (Feng, 2023; Watts et al., 2023). Some endophytes can produce substances that are beneficial to the host to help the host resist the harm caused by changes in the external environment (Kashyap et al., 2023). The endophytes that can help the host resist the harm caused by pathogenic bacteria are biocontrol endophytes. Different types of biocontrol bacteria play different roles. Some produce various hormones to promote plant growth, whereas others directly promote the acquisition of nutrients by the host or enhance the tolerance of the host to abiotic stress (Khalaf and Raizada, 2018; Chu and Bae, 2022). Some bacteria can produce antibacterial metabolites to inhibit the growth and reproduction of foreign microorganisms and even engulf foreign microorganisms or directly stimulate the host’s defense mechanism to remind the host to defend against enemies (Bastías and Gundel, 2023; Waqar et al., 2024). Lu et al. isolated fifty candidate strains from camphor-tree, of which 11 strains were antagonistic to canker pathogens of *Fusiccocum* sp. and *Phomopsis* sp (Lu et al., 2018). Fan found that the endophytic bacteria of the genus *Constrictibacter* and the endophytic fungi of the genus *Chaetomidium* in *Populus tomentosa* were significantly enriched after being infected with the *populus* decay disease (Fan, 2023). Obviously, there exists a complex interaction relationship among endophytes, plants and the environment, which plays an important role in plant growth and stress resistance. As an important type of microbial resource, endophytes have also received increasing attention in the biological control of plant diseases.

With growing awareness of ecological protection and environmental safety, using pollution-free biological methods to control plant diseases and pests has attracted increasing attention (Kashyap et al., 2023). To date, research on *Idesia polycarpa* Maxim. has largely focused on its fruit and oil, while fewer studies have addressed antagonistic microorganisms against stem rot in this tree species. The seriousness of the disease causes numerous tree deaths, impacts seedling production and fruit yield, and constrains the development of the *I. polycarpa* industry. Accordingly, we hypothesized that endophytes isolated from healthy *I. polycarpa* plants could produce active metabolites that inhibit the growth of *B. dothidea*, and some strains possessed potential biocontrol activity against the stem canker pathogen. To test this hypothesis, we isolated endophytes from healthy *I. polycarpa* and conducted a systematic screening based on antagonistic activity, molecular identification, *in vivo* biocontrol efficacy assays, and biological characterization. As a result, an endophytic biocontrol bacterium, designated B47, was obtained, which exhibits obvious antagonism against the stem rot pathogen.

In this study, endophytes were isolated and purified from healthy *I. polycarpa* Maxim., and those with antagonistic activity against *B. dothidea* were screened for potential use as biocontrol agents. This approach offers a new direction for the management of stem rot in *I. polycarpa* and holds substantial importance for reducing chemical use and protecting the environment. This will provide new direction for the prevention and treatment of *I. polycarpa* stem canker disease and promote the high-quality development of the *I. polycarpa* industry.

## 2 Materials and methods

### 2.1 Testing material

We collected new leaves, bracts, tender stems and fruits from healthy ten-year-old *I. polycarpa* trees, and young roots from one-year-old seedlings as materials for endophyte isolation. Afterwards, all samples were pretreated and surface-disinfected. They were collected from the Science and Education Park of Henan Agricultural University (34°86′N and 113°59′E) and the Forest Seedling Breeding Engineering Technology Center of Henan Agricultural University (34°48′N and 113°38′E) Henan, China. The *I. polycarpa* in this study was the provincial-level certified superior variety ‘Yuji’, which was selected and bred by our research team. *B. dothidea* was previously isolated from naturally diseased *I. polycarpa* plants (Fang et al., 2024). This strain was verified to be a stem canker pathogen of *I. polycarpa* and was stored at 4 °C in the Laboratory of Forest Physiology and Ecology of Henan Agricultural University in China. The isolation of endophytes from *Idesia polycarpa* was initiated in April 2023.

The culture media used in this study included Potato Dextrose Agar (PDA) (02-023, Beijing Aoboxing Biotechnology Co., Ltd., China), Beef Extract Peptone Solid Medium (NA) (02-275, Beijing Aoboxing Biotechnology Co., Ltd., China), and Beef Extract Peptone Liquid Medium (NB) (02-013, Beijing Aoboxing Biotechnology Co., Ltd., China). All media were supplied by Henan Huafeng Chemical Reagent Co., Ltd.

The main chemical reagents included a rapid fungal genomic DNA extraction kit (B518229, Sangon Biotech Engineering (Shanghai) Co., Ltd., China, 100 preps), a rapid bacterial genomic DNA extraction kit (B518225, Sangon Biotech Engineering (Shanghai) Co., Ltd., China, 100 preps), 75% ethanol (CAS: 64-17-5, Sinopharm Chemical Reagent Co., Ltd., China), 2% sodium hypochlorite (CAS: 7681-52-9, Sinopharm Chemical Reagent Co., Ltd., China), β-mercaptoethanol (CAS: 60-24-2, Shanghai Macklin Biochemical Technology Co., Ltd., China), isopropanol (CAS: 67-63-0, Shanghai Macklin Biochemical Technology Co., Ltd., China), *Taq* DNA polymerase and its buffer (cat. T917854, Shanghai Macklin Biochemical Technology Co., Ltd., China), and dNTPs (cat. N917488, Shanghai Macklin Biochemical Technology Co., Ltd., China) were supplied by Henan Huafeng Chemical Reagent Co., Ltd.

The principal instruments used included an electronic balance (FA2204C, Shanghai Tianmei Balance Instrument Co., Ltd., China, 0.1 mg/220 g), a clean bench (SW−CJ−1D, Suzhou Antai Air Tech Co., Ltd., China, ISO Class 5/vertical laminar flow), a fume hood (TF−1500, Shanghai Yiheng Scientific Instrument Co., Ltd., China), a PCR thermal cycler (Veriti96, Thermo Fisher Scientific, USA), an electrophoresis apparatus (DYY−6C, Beijing Liuyi Biotechnology Co., Ltd., China), a high−speed refrigerated centrifuge (HC−3018R, Anhui Zhongke Zhongjia Scientific Instrument Co., Ltd., China), a vertical automatic electrothermal pressure steam sterilizer (LDZX−50KBS, Shanghai Shenan Medical Instrument Factory, China), an intelligent biochemical incubator (LRH−250, Shanghai Yiheng Scientific Instrument Co., Ltd., China), a modulated fluorescence analyzer (FMS−2, Hansatech Instruments, UK), and a shaker (THZ−82A, Changzhou Guohua Electric Appliance Co., Ltd., China), among others.

### 2.2 Isolation and purification of endophytes

Isolation of endophytic fungi: tissues including new leaves, bracts, tender stems and fruits were sampled from ten-year-old healthy *I. polycarpa* trees, and young roots were collected from one-year-old seedlings. All samples were washed with running water for 30 min, and the water was completely removed with absorbent paper. The 0.5 cm × 0.5 cm samples were cut at the appropriate positions on the leaves (the tender stems were cut into 1 mm thin slices horizontally, the bracts and fruits were cut in half, and the rhizomes were cut into 0.5 cm pieces each). On the ultraclean bench, the samples were immersed in 75% alcohol for 30 s, soaked in 2% sodium hypochlorite for 1 min, rinsed with sterile water 3 times, and dried with sterile filter paper. The samples were inoculated on PDA media with tweezers after high-temperature disinfection. Each medium was inoculated with 4 samples, each sample was inoculated with 3 media, and the sterile water that had been rinsed for the last time was used as the control. The inoculated media were placed in an intelligent biochemical incubator and cultured in the dark at 28 °C. The number, color and shape of the colonies on the media were observed once a day. Endophytic fungi were isolated and cultured using the routine method that is currently employed for the majority of endophytic fungi, following the detailed protocols published by Huang et al. (2025).

Isolation of endophytic bacteria: the surface disinfection regimen applied to endophytic bacteria was the same as that for endophytic fungi. All disinfected samples were placed into a sterilized mortar and ground. On the ultraclean bench, after adding 5 mL of sterile water, 1 mL of the resulting plant tissue homogenate was taken and separately diluted 10−fold, 100−fold, and 1000−fold with sterile water. The initial homogenate and its serial dilutions were each spread onto NA media using a sterilized inoculation loop. For each concentration of every sample, three replicate culture media were inoculated, with the final rinse sterile water serving as the blank control. The inoculated media were incubated in an intelligent biochemical incubator at 28 °C in the dark. The number, color, and morphology of colonies on the media were examined daily. The isolation protocol for endophytic bacteria also referred to Huang et al. (2025).

Purification of endophytes: The fungi and bacteria grown on the primary culture media were respectively inoculated onto fresh potato dextrose agar (PDA) and nutrient agar (NA) media. This purification procedure was repeated until homogeneous single colonies were obtained, and the purified strains were preserved on slant media for subsequent use. Subsequently, the antibacterial activities of the purified endophytes were assayed.

### 2.3 Screening of biocontrol endophytes

The laboratory-preserved pathogen *Botryosphaeria dothidea* and the 179 isolated endophytic strains were revived and subjected under dark conditions at 28 °C. After 5 days, fresh agar discs (5 mm in diameter) were punched from the colony margins using a sterile cork borer for subsequent use.

Preliminary screening of biocontrol endophytes: a fresh 5 mm diameter sample of *B. dothidea* was inoculated at the center of the PDA plate, and four 5 mm diameter endophytes were inoculated equidistantly at a distance of 2.5 cm from the *B. dothidea* sample. The PDA plate that was inoculated with *B. dothidea* alone was used as a control and was cultured at 28 °C for 7 days. Each endophyte had 3 replicates. The radius of the pathogen colony was measured and recorded every day. The biocontrol agents exhibiting antagonistic activity against *Botryosphaeria dothidea* were preliminarily screened.

Rescreening of biocontrol endophytes: a fresh 5 mm-diameter *B. dothidea* cake and an antagonistic endophytic cake were inoculated at both ends of a PDA plate with the same diameter, and the contact point was 2 cm away from the edge of the medium. A PDA plate that was inoculated with a *B. dothidea* cake alone was used as a control and was cultured in the dark at 28 °C for 7 days. The colony radius of the pathogen in the control group and the directional radius in confrontation culture were measured along two perpendicular diameters, and the inhibition rate was calculated accordingly. The distance from the edge of the colony to the center of the control group was R1; the distance from the edge to the center of *B. dothidea* colony in the experimental group was R2; the distance from the edge of *Botryosphaeria* colony to the edge of an adjacent fungus colony is R3., that is, the bacteriostatic radius. R3 (%) = (R1 – R2)/R1 × 100%. Each antagonistic strain had 3 replicates.

### 2.4 Identification of biocontrol endophytes

Seven biocontrol endophytes were selected for molecular identification: five fungal strains (A5, A7, A17, A18, A22) and two bacterial strains (B47 and B55). DNA was extracted according to the instructions of the fungal genomic DNA rapid extraction kit (B518229, Sangon Biotech Engineering (Shanghai) Co., Ltd., China, 100 preps) and the bacterial genomic DNA rapid extraction kit (B518225, Sangon Biotech Engineering (Shanghai) Co., Ltd., China, 100 preps), For Gram−positive bacterial strains (B47 and B55), an additional lysozyme incubation step (180 μL lysozyme solution at 37 °C for 30 min) was performed before digestion. Extracted DNA was stored at -20 °C.

The extracted genomic DNA was used as a template for PCR amplification. The universal fungal primers used were ITS1 (5’-TCCGTAGGTCCTGCGG-3’) and ITS4 (5’-TCCTCCGCTTATTGATATGC-3’) (Zhang, 2020). The PCR system (25 μL) included 2.5 μL of 10x PCR buffer, 1 μL of 10 mmol/L dNTPs, 1 μL of 10 μmol/L primer, 0.5 μL of 5U/μL Taq DNA polymerase, 1.0 μL of DNA template, and 18 μL of ultrapure water. The universal primers used for bacterial DNA amplification were 27F (5’-AGAGTTTGATCCTGGCTCAG-3’) and 1525R (5’-AAGGAGGTGATCCAGCCGCA-3’) (Song, 2018). The PCR system (25 μL) included 2.5 μL of 10×PCR buffer, 2.0 μL of 25 mmol/L MgCl2, 0.5 μL of 10 mmol/L dNTP, 0.2 μL of 5 U/μL Taq DNA polymerase, 2 μL of 10 μmol/L primer, 1 μL of DNA template, and 14.8 μL of ultrapure water. After the reaction was complete, the PCR products were detected by electrophoresis on 1.0% agarose gel, and the PCR products were recovered and sent to a company for sequencing.

### 2.5 Inoculation of *in vitro* branches with biocontrol endophytes

One-year-old branches of healthy *I. polycarpa* were selected, which were approximately 1 cm thick, cut into appropriate sizes, cleaned with clean water and medical absorbent cotton, disinfected with 75% alcohol for 2 min, rinsed with running water to remove alcohol, and rinsed with sterile water once. Both two ends of each branch were sealed with paraffin, and an appropriate size was removed with a sterile puncher at the appropriate position and placed in a closed plastic box. The two ends of the branch were cushioned with sterile absorbent cotton. In the *in vitro* branch inoculation experiment, four treatments were set up: a control (CK0), inoculated with only blank medium; DZ1, inoculated with only *B. dothidea* cake; DZ2, biocontrol endophytes cake and *B. dothidea* cake inoculated together; and DZ3, inoculated with only biocontrol endophytes cake. Each treatment had 3 replicates. After inoculation, the plastic baskets were sealed with plastic wrap, placed in a climate box, cultured at 28 °C, and sprayed with sterile water three times a day. After 10 days, the phloem of the branches was scraped, the lesion lengths were measured, and the disease index and relative control effect were calculated according to the disease index table (**Table 1**), formula of the disease index and relative control effect (Li et al., 2023). Disease index (%) = Σ [(the representative value of this level × the number of diseased plants)/(the number of trees surveyed × the highest representative value)] × 100%. Relative control effect (%) = (control disease index – treatment disease index)/control disease index × 100%.

**Table 1:** Grading criteria for disease index of stem canker caused by *Botryosphaeria dothidea* on *Idesia polycarpa*.

Lesion diameter (cm)	Grade	Representative value
0	〇	0
0 < d ≤ 0.5	I	1
0.5 < d ≤ 1.0	II	2
1.0 < d≤ 1.5	III	3
1.5 < d ≤ 2.0	IV	4
d > 2.0	V	5

### 2.6 Plant experiment involving biocontrol endophytes

Two-year-old *I. polycarpa* seedlings (with a basal stem of approximately 8 mm diameter) with similar, healthy growth conditions were selected, and the appropriate sizes were removed with a sterile puncher at the appropriate positions on the stems to carry out the *in vivo* plant inoculation test. Three treatments were used in the experiment: control (CK1), inoculated with only blank medium; DY1, inoculated with only *B. dothidea* cake; and DY2, co-inoculation with biocontrol endophytes cake and *B. dothidea* cake. Each treatment group had 10 replicates. The inoculation sites were wrapped with sterile absorbent cotton and sprayed with sterile water to keep them wet. After 30 days, the growth of the calli at the inoculation sites was observed, and the calli and phloem at the wounds were removed. The length of the xylem lesions was measured, and the disease index and relative control effect were calculated according to the disease index table (**Table 1**), formula of the disease index and relative control effect (Li et al., 2023).

### 2.7 Biological characteristics of biocontrol endophytes

For the sugar fermentation experiment, the media used were as follows: (NH_4_)_2_HPO_4_, 1.0 g; MgSO_4_, 0.2 g; KCl, 0.2 g; yeast extract, 0.2 g; glucose, 10 g; agar, 5 g; and 0.04% bromophenol blue, 20 mL. The pH was adjusted to 7.0-7.2, distilled water was added to a constant volume of 1 000 mL, and the mixture was placed in a three-dimensional electrothermal pressure steam sterilizer for sterilization at 121 °C for 20 min. The biocontrol endophytes that were inoculated on the NA medium were cultured overnight, and the biocontrol endophytes were inoculated on the medium required for the test with the inoculation ring and were placed in the intelligent biochemical incubator at 28 °C in the dark. If biocontrol endophytes produced acid, the medium changed from blue to yellow.

For the catalase test, the biocontrol endophytes that were inoculated on NA media were cultured overnight, and the inoculated ring was coated on a clean glass slide. One drop of 3% hydrogen peroxide solution was added to observe whether bubble formation occurred within 30 s. A positive result indicated that the biocontrol endophytes were positive, and vice versa.

For the aerobic determination and exercise test, the following media were used: peptone, 2 g; NaCl, 5 g; K_2_HPO_4_, 0.2 g; agar, 5 g; glucose, 10 g; and distilled water, 1 000 mL, and a three-dimensional electric pressure steam sterilization pot that was sterilized at 121 °C for 20 min was used. The medium was poured into a sterilized plug-type test tube, cooled and solidified. The biocontrol endophytes that were cultured overnight were inoculated into the bottom of the test tube with the inoculation ring and cultured in an intelligent biochemical incubator at 28 °C in the dark. The results were observed after 3 days. If the biocontrol endophytes grew on the surface of the medium, the biocontrol endophytes were considered aerobic and positive; if the biocontrol endophytes grew along the solid line, the biocontrol endophytes were considered anaerobic and negative.

For the methyl red test, the following media were used: 5 g of K_2_HPO_4_, 5 g of peptone, and 5 g of glucose. The pH was adjusted to between 7.0-7.2, distilled water was added to a level of 1 000 ml, and the mixture was placed into a three-dimensional electric pressure steam sterilization pot for sterilization at 121 °C for 20 min. The required reagent configuration was as follows: 0.1 g of methyl red, 300 mL of 95% alcohol, and 500 mL of distilled water. The biocontrol endophytes were inoculated into NB medium and cultured overnight on a shaker. After inoculation into the medium required for the test at a volume ratio of 5%, the mixture was cultured at 25 °C in a shaker at 180 r/min. One drop of methyl red reagent was added to the culture medium on the 2nd, 4th, and 6th days of culture. If the culture medium became red, this result indicated that the biocontrol endophytes were positive. If the culture medium became yellow, this result indicated that the biocontrol endophytes were negative.

Determination of growth curves: the biocontrol endophytes were inoculated into NB medium for overnight culture on a shaker, inoculated into new NB medium at a volume ratio of 5%, and cultured at 25 °C in a shaker at 180 r/min. The OD600 values were measured at 0, 0.5, 1, 1.5, 2, 2.5, 3, 4, 5, 6, 8, 10, 12, 14, 16, 18, 20, 24, 28, 32, 48 and 72 h. Three replicates were defined for each time point, and NB medium without inoculation was used as a blank control.

Determination of salt tolerance: the biocontrol endophytes were inoculated into NB medium overnight on a shaker and inoculated with NB medium containing 0%, 2%, 5%, 7%, or 10% NaCl at a volume ratio of 5%. At the same time, the OD600 value of the medium was determined as that of the control group. The medium was placed in a 180 r/min shaker at 25 °C for 24 h, and the OD600 values were measured again, with 3 replicates for each concentration. The uninoculated NB medium was used as the blank control when the OD600 values were measured.

Determination of the optimum growth temperature: the biocontrol endophytes were inoculated into NB medium and cultured overnight on a shaker. They were inoculated into new NB medium at a volume ratio of 5% and placed in a constant-temperature water bath. The OD600 values of the media after 0 h and 24 h were measured. The OD600 value at 0 h represented that of the control group at 24 h. The temperatures were 5, 10, 15, 20, 25, 30, 35, 40, 45 and 50 °C. Three replicates were set for each temperature, and NB medium without inoculation was used as the blank control.

Determination of the optimum growth pH value: the biocontrol endophytes were inoculated into the NB medium overnight on a shaker and then inoculated into the NB medium at pH values of 3, 4, 5, 6, 7, 8, 9, 10, and 11 at a volume ratio of 5%. The pH value of NB medium was adjusted by HCl and NaOH solution. At the same time, the OD600 value of the medium was determined as that of the control group. The culture media were placed in a 180 r/min shaker at 25 °C for 24 h, and the OD600 values were measured. Three replicates were set for each concentration, and the uninoculated NB medium was used as the blank control.

### 2.8 Statistical analysis

All experiments were performed with three independent replicates. These data obtained from the experiments were analyzed by variance analysis using SPSS 17.0 (SPSS Inc., Chicago, IL, USA), and Duncan’s test was used for multiple comparisons (*p* < 0.05). The results of 16SrDNA gene sequencing were compared with the nucleic acid sequence in the NCBI database (https://blast.ncbi.nlm.nih.gov/Blast.cgi), and a phylogenetic tree was constructed by MEGA7.0 software.

## 3 Results

### 3.1 Separation and purification of strains

According to the differences in colony morphology and color on PDA and NA solid media, 179 endophytic strains were isolated, including 105 fungi strains and 74 bacteria strains (**Table 2**). The number of endophytes that were isolated from the buds and rhizomes was the greatest, and that from the fruit was the lowest. Different kinds of endophytes coexist in different parts of *I. polycarpa* plants.

**Table 2:** Isolation results of endophytes from different tissue parts of *Idesia polycarpa*.

Specimen	Leaf	Flower	Stem	Fruit	Root	Total
Fungus strains	37	43	12	5	8	105
Bacterial strains	12	9	9	0	44	74
Total	49	52	21	5	52	179

### 3.2 Screening of biocontrol endophytes

**Figure 1:**
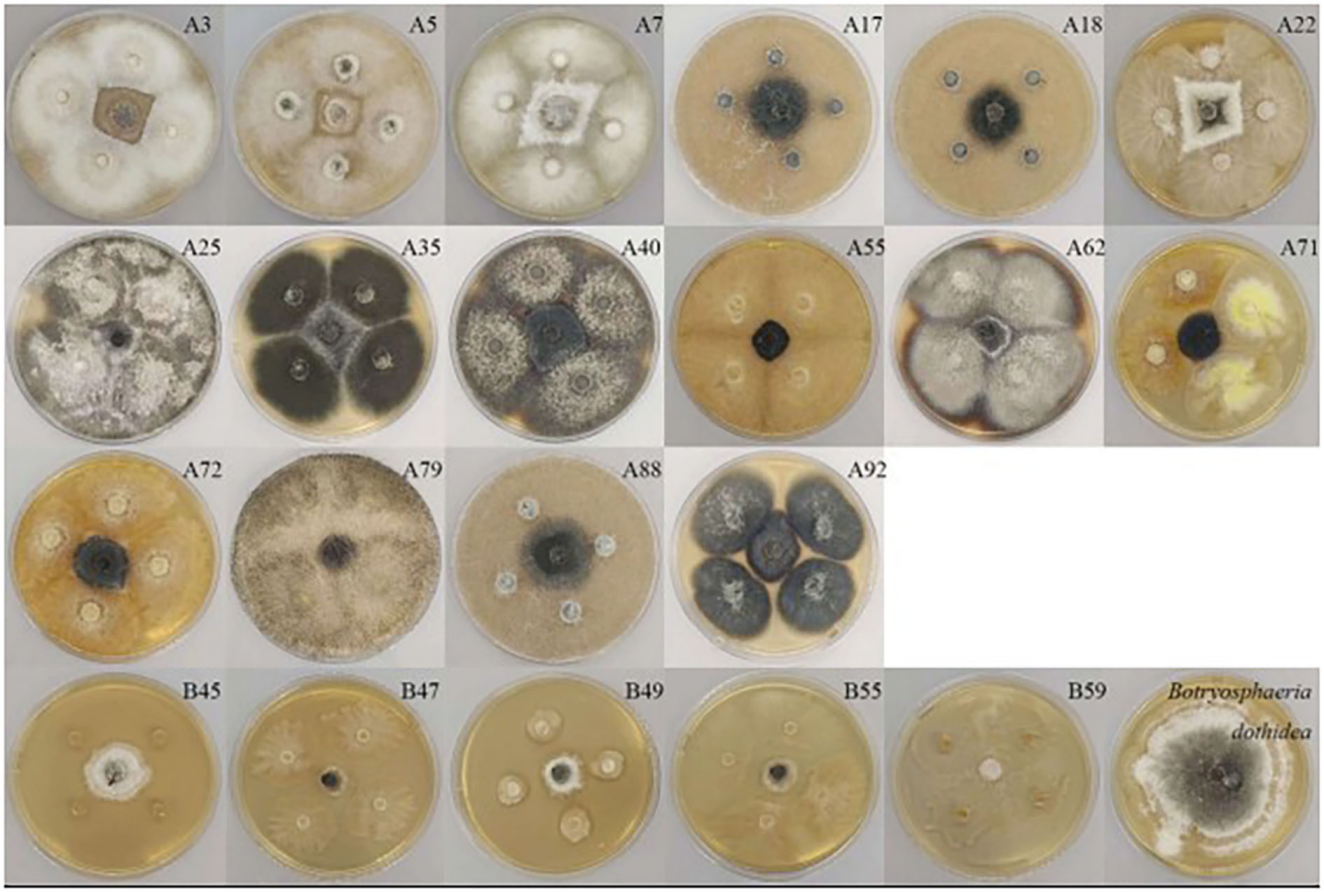
Antimicrobial effect of endophytic strains with an inhibition rate of more than 50% against *B. dothidea* via five-point confrontation method. A3, A5, A7, A17, A18, A22, A25, A35, A40, A55, A62, A71, A72, A79, A88 and A92 represented endophytic fungi, while B45, B47, B49, B55 and B59 represented endophytic bacteria, the rightmost figure in the fourth row showed the *B. dothidea*.

**Figure 2:**
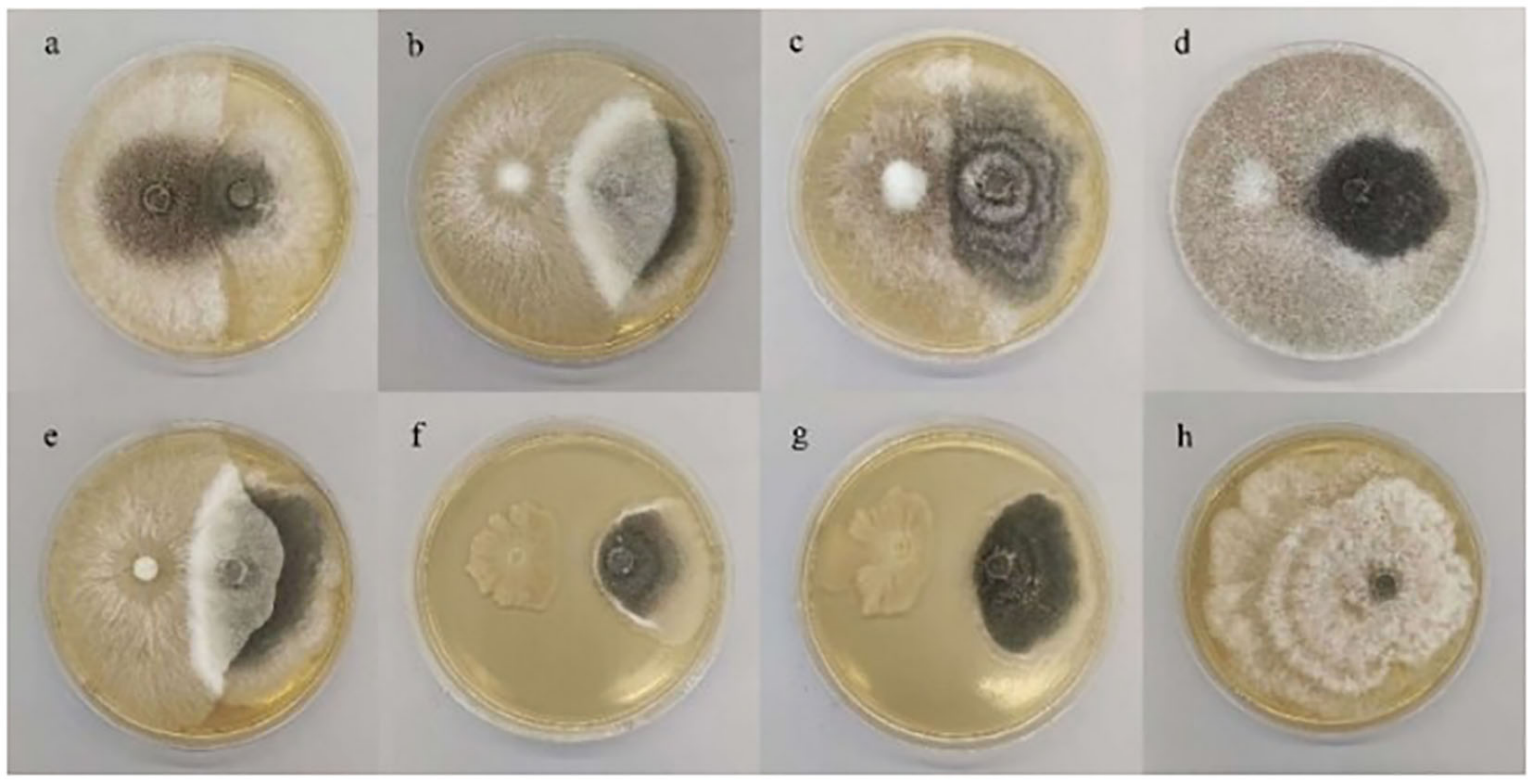
Antimicrobial effect of endophytic strains with an inhibition rate of more than 75% against *B. dothidea* via two-point confrontation method. **(a–e)** were endophytic fungi A5, A7, A17, A18 and A22, respectively. While **(e, f)** were endophytic bacteria B45 and B55. **(h)** was the *B. dothidea*.

The five-point confrontation method was employed to screen 39 endophytic fungal strains and 11 endophytic bacterial strains (**Table 3**) (*p*<0.05). Among the endophytes with inhibition rates greater than 50%, 16 fungal strains and 5 bacteria strains were identified (**Figure 1**). The two-point plate confrontation method was used for rescreening, 33 fungal strains and 3 bacterial strains with more than 50% inhibition rates were identified (**Table 4**) (*p*<0.05). Among these strains 5 fungal strains and 2 bacterial strains had inhibition rates greater than 75% (**Figure 2**). A5 (inhibition rate: 76.07% ± 5.18) was isolated from stems, A7 (inhibition rate: 83.01% ± 2.90), A17 (inhibition rate: 75.38% ± 5.72) and A18 (inhibition rate: 75.38% ± 5.72) were isolated from leaves, while A22 (inhibition rate: 82.14% ± 1.24), B47 (inhibition rate: 84.56% ± 1.00) and B55 (inhibition rate: 84.45% ± 2.16) were isolated from roots.

**Table 3:** Inhibition rates of endophytic strains against *Botryosphaeria dothidea* in the preliminary screening using the five-point confrontation method (mean ± SD, n=3).

Type	Strain	Average diameter (mm)	Antibacterial rate (%)	Strain	Average diameter (mm)	Antibacterial rate (%)
Endophytic fungi	A1	4.48 ± 0.37 abcdef	39.18 ± 8.40 abcd	A56	7.02 ± 0.46 a	4.57 ± 13.24 d
	A2	3.68 ± 0.83 abcdef	49.87 ± 13.15 abcd	A60	5.23 ± 1.43 abcdef	27.21 ± 25.09 abcd
	A3	2.62 ± 0.06 cdef	64.46 ± 4.15 abcd	A61	4.52 ± 0.62 abcdef	38.91 ± 9.97 abcd
	A5	2.05 ± 0.08 def	72.11 ± 3.70 ab	A62	2.65 ± 0.37 cdef	64.37 ± 4.27 abcd
	A7	1.97 ± 0.17 f	73.16 ± 4.66 a	A71	3.12 ± 0.88 bcdef	56.67 ± 15.56 abcd
	A13	5.88 ± 0.31 abcde	20.36 ± 7.71 abcd	A72	3.20 ± 0.65 abcdef	56.17 ± 12.00 abcd
	A17	2.90 ± 0.55 bcdef	59.98 ± 10.85 abcd	A77	3.60 ± 1.56 abcdef	49.86 ± 25.37 abcd
	A18	2.65 ± 0.58 cdef	64.44 ± 7.06 abcd	A79	2.03 ± 0.35 ef	72.16 ± 6.49 ab
	A20	4.17 ± 2.15 abcdef	42.97 ± 31.39 abcd	A80	3.98 ± 0.35 abcdef	46.33 ± 4.01 abcd
	A22	2.28 ± 0.21 cdef	69.26 ± 1.89 abc	A88	3.55 ± 0.23 abcdef	51.90 ± 5.36 abcd
	A23	3.72 ± 1.26 abcdef	48.48 ± 21.39 abcd	A89	4.77 ± 0.73 abcdef	35.17 ± 12.83 abcd
	A25	2.10 ± 0.15 def	71.62 ± 2.62 ab	A90	6.05 ± 0.41 abc	18.21 ± 7.84 abcd
	A26	4.78 ± 2.02 abcdef	37.59 ± 19.85 abcd	A91	3.95 ± 0.40 abcdef	45.94 ± 10.31 abcd
	A34	4.80 ± 1.82 abcdef	33.58 ± 30.03 abcd	A92	3.28 ± 0.27 abcdef	55.50 ± 5.62 abcd
	A35	3.08 ± 0.06 bcdef	58.28 ± 3.31 abcd	A94	5.48 ± 2.17 abcdef	24.14 ± 35.43 abcd
	A37	3.97 ± 1.17 abcdef	45.05 ± 20.45 abcd	A99	5.97 ± 0.95 abc	17.89 ± 19.92 abcd
	A39	3.70 ± 1.15 abcdef	49.03 ± 19.48 abcd	A100	6.67 ± 0.85 ab	8.53 ± 19.60 bcd
	A40	3.53 ± 0.77 abcdef	51.28 ± 14.60 abcd	A101	5.90 ± 0.18 abcd	19.94 ± 8.78 abcd
	A41	4.95 ± 0.50 abcdef	32.54 ± 11.95 abcd	A103	6.72 ± 1.69 ab	7.09 ± 29.17 cd
	A55	2.22 ± 0.39 cdef	69.53 ± 7.52 abc			
Endophytic bacteria	B41	6.42 ± 1.11 a	12.09 ± 20.67 c	B50	5.42 ± 0.70 b	27.04 ± 8.93 ab
	B44	6.28 ± 1.80 a	17.08 ± 16.43 c	B55	2.00 ± 0.57 ab	73.59 ± 4.74 bc
	B45	2.22 ± 0.48 b	70.57 ± 3.41 ab	B56	5.05 ± 0.20 ab	31.36 ± 8.61 abc
	B46	5.37 ± 1.27 ab	28.71 ± 10.57 bc	B57	6.32 ± 1.78 a	12.71 ± 29.26 c
	B47	1.45 ± 0.15 b	80.24 ± 3.32 a	B59	3.08 ± 1.86 ab	60.49 ± 19.74 abc
	B49	2.05 ± 0.12 b	72.37 ± 1.27 ab			
Pathogenic bacteria	*B. dothidea*	7.45 ± 0.76				

Different lowercase letters indicate statistically significant differences (*p* < 0.05).

**Table 4:** Inhibition rates of endophytic strains with more than 50% inhibitory activity against *Botryosphaeria dothidea* in the secondary screening using the two-point confrontation method (mean ± SD, n=3).

Type	Strain	Average diameter (mm)	Antibacterial rate (%)	Strain	Average diameter (mm)	Antibacterial rate (%)
Endophytic fungi	A1	1.50 ± 0.16 bcdef	69.44 ± 5.50 abc	A41	1.63 ± 0.37 bcde	67.32 ± 6.14 abc
	A2	1.53 ± 0.05 bcdef	68.82 ± 4.52 abc	A55	2.40 ± 0.08 a	51.55 ± 3.94 c
	A3	1.43 ± 0.05 bcdefg	70.96 ± 3.33 ab	A61	1.33 ± 0.09 cdefg	67.46 ± 1.60 ab
	A5	1.17 ± 0.12 efg	76.07 ± 5.18 ab	A62	1.60 ± 0.08 bcde	58.39 ± 5.01 abc
	A7	0.83 ± 0.05 g	83.01 ± 2.90 a	A71	2.03 ± 0.45 ab	60.43 ± 11.04 bc
	A17	1.20 ± 0.14 defg	75.38 ± 5.72 ab	A72	1.97 ± 0.12 abc	74.44 ± 2.07 bc
	A18	1.20 ± 0.14 defg	75.38 ± 5.72 ab	A77	1.23 ± 0.25 defg	74.59 ± 7.80 ab
	A20	1.43 ± 0.17 bcdefg	70.82 ± 5.76 ab	A79	1.27 ± 0.17 defg	63.79 ± 2.50 ab
	A22	0.90 ± 0.16 fg	82.14 ± 1.24 a	A80	1.80 ± 0.14 abcde	74.40 ± 2.39 bc
	A23	1.37 ± 0.12 cdefg	72.38 ± 3.61 ab	A88	1.27 ± 0.12 defg	62.94 ± 3.44 ab
	A25	1.40 ± 0.00 bcdefg	71.63 ± 3.28 ab	A89	1.83 ± 0.05 abcd	71.04 ± 3.56 bc
	A26	1.40 ± 0.08 bcdefg	71.82 ± 1.60 ab	A91	1.43 ± 0.05 bcdefg	69.95 ± 2.64 ab
	A34	1.57 ± 0.09 bcde	68.27 ± 3.85 abc	A92	1.50 ± 0.22 bcdef	73.54 ± 2.89 ab
	A35	1.63 ± 0.33 bcde	67.53 ± 3.61 abc	A94	1.30 ± 0.08 defg	68.24 ± 4.34 ab
	A37	1.57 ± 0.12 bcde	68.32 ± 3.98 abc	A99	1.57 ± 0.05 bcde	66.69 ± 3.93 abc
	A39	1.37 ± 0.12 cdefg	72.51 ± 2.24 ab	A103	1.63 ± 0.12 bcde	67.46 ± 6.10 abc
	A40	1.40 ± 0.08 bcdefg	71.54 ± 4.13 ab			
Endophytic bacteria	B47	0.77 ± 0.05 b	84.56 ± 1.00 a	B55	0.77 ± 0.05 b	84.45 ± 2.16 a
	B50	2.13 ± 0.05 a	56.77 ± 4.93 ab			
Pathogenic bacteria	*B. dothidea*	5.00 ± 0.57				

Different lowercase letters indicate statistically significant differences (*p* < 0.05).

### 3.3 Identification of biocontrol endophytic strains

**Figure 3:**
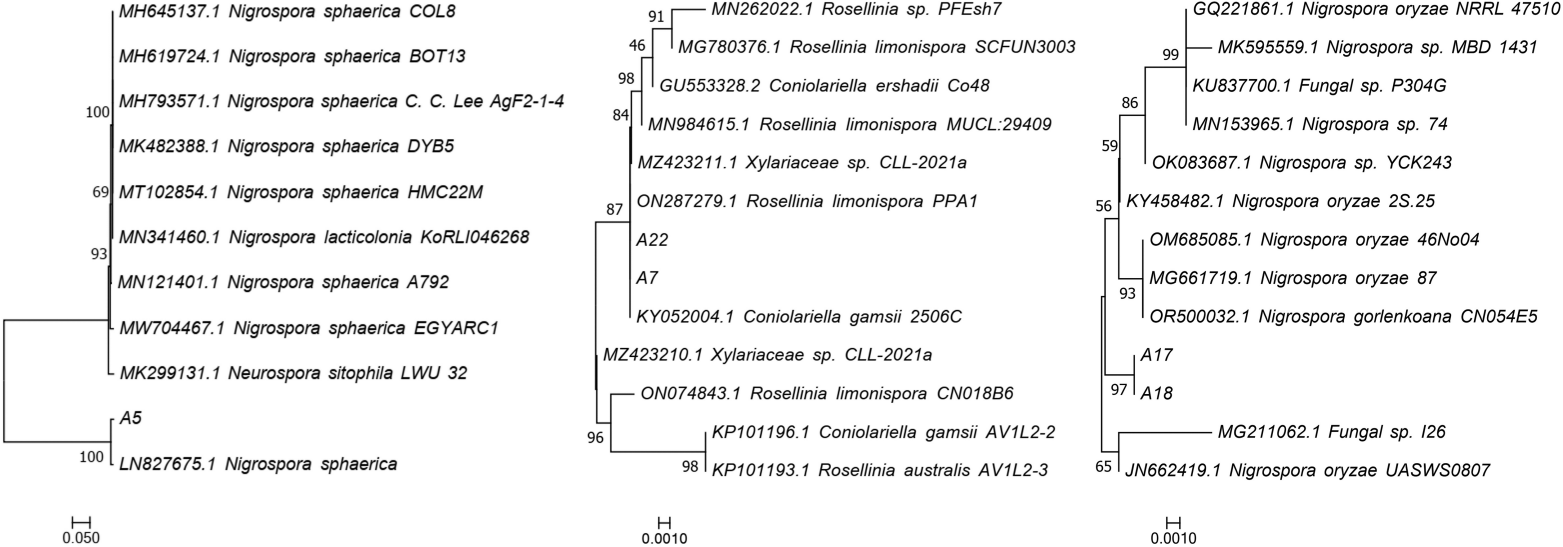
Phylogenetic tree of endophytic fungi A5, A7, A17, A18 and A22 from *I. polycarpa*. The value of the node position represents the confidence, which reflects the reliability of the branch represented by the node in the evolutionary relationship. The branch length represents the degree of change during evolution.

**Figure 4:**
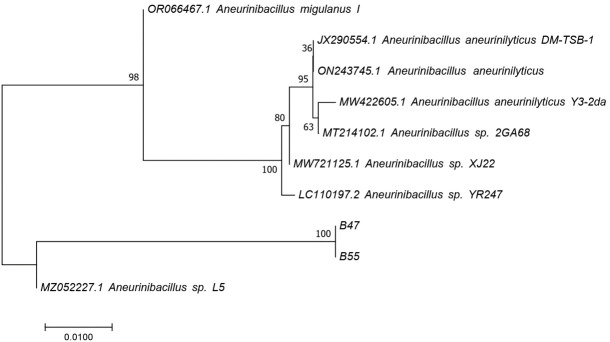
Phylogenetic tree of endophytic bacteria B47 and B55 from *I. polycarpa*. The value of the node position represents the confidence, which reflects the reliability of the branch represented by the node in the evolutionary relationship. The branch length represents the degree of change during evolution.

The results of gram staining of the endophytic bacteria, B47 and B55, were positive, and lysozyme was added to extract their DNA. Sequencing was performed on the PCR products of seven endophytic strains (A5, A7, A17, A18, A22, B47, B55) exhibiting high antagonistic activity. Homology searches were subsequently conducted using the NCBI database. By comparing the similarities, the genus and species of each endophyte were determined (**Figures 3**, **4**). The similarity between A5 and *Nigrospora lacticolonia* MM4-1z4 B was 99.78%. The DNA sequences of A17 and A18 were the same, and their similarities with *Nigrospora oryzae* Nory01-18 were 100%. The DNA sequences of A7 and A22 were the same, and their similarities with *Rosellinia limonispora* MUCL: 29409, were 100%. Therefore, the endophytic fungi, A5, A7, A17, A18, and A22 were not suitable for further research as effective biocontrol bacteria against endophytic antagonistic strains of *B. dothidea*. The DNA sequences of the endophytic bacteria, B47 and B55, were the same, with 96.92% similarity to *Aneurinibacillus* sp. YR247, 97.58% similarity to *Aneurinibacillus migulanus* I, 97.74% similarity to *Aneurinibacillus migulanus* 25, and 97.83% similarity to *Aneurinibacillus* sp. L5. Phylogenetic analysis indicated that strain B47 clustered within the same clade as strains of the genus *Aneurinibacillus*. Based on the aforementioned sequence similarity and phylogenetic position, B47 was identified as an undetermined species of the genus *Aneurinibacillus* and was named as *Aneurinibacillus* sp. B47.

### 3.4 Inoculation of shoots *in vitro*

**Figure 5:**
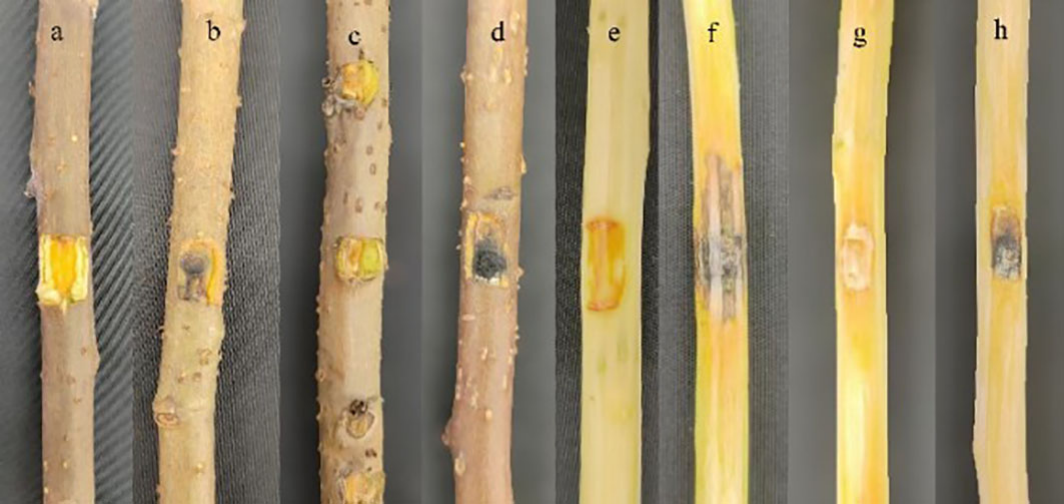
The situation of endophytic bacteria B47 and *B. dothidea* being inoculated on isolated branches of *I. polycarpa* for 15 days. **(a, e)** represented CK, **(b, f)** represented single inoculations of *B. dothidea*, **(c, g)** represented single inoculations of endophytic bacteria B47, and **(e, h)** represented simultaneous inoculations of *B. dothidea* and endophytic bacteria B47. After excluding the inoculation length of the mycelial plug, the lesion length of group e was 1.97 ± 0.70, and that of group f was 1.18 ± 0.37.

The results of *in vitro* branch analysis after 10 days of inoculation were shown in **Figure 5**. Calli grew from the inoculation sites of *in vitro* branches that were inoculated with B47 alone, which were similar to those of the CK group, and the branches presented high activity, indicating that B47 was harmless to *I. polycarpa* plants. The branches that were inoculated with *B. dothidea* alone presented lesions, and the disease index was 84%. Co-inoculation with *B. dothidea* and strain B47 reduced the disease index to 48%, with a relative inhibition rate of 42.86%, demonstrating that the endophytic bacterium B47 can suppress the growth and dissemination of *B. dothidea* on detached branches.

### 3.5 Plant experiment involving biocontrol of endophytes

**Figure 6:**
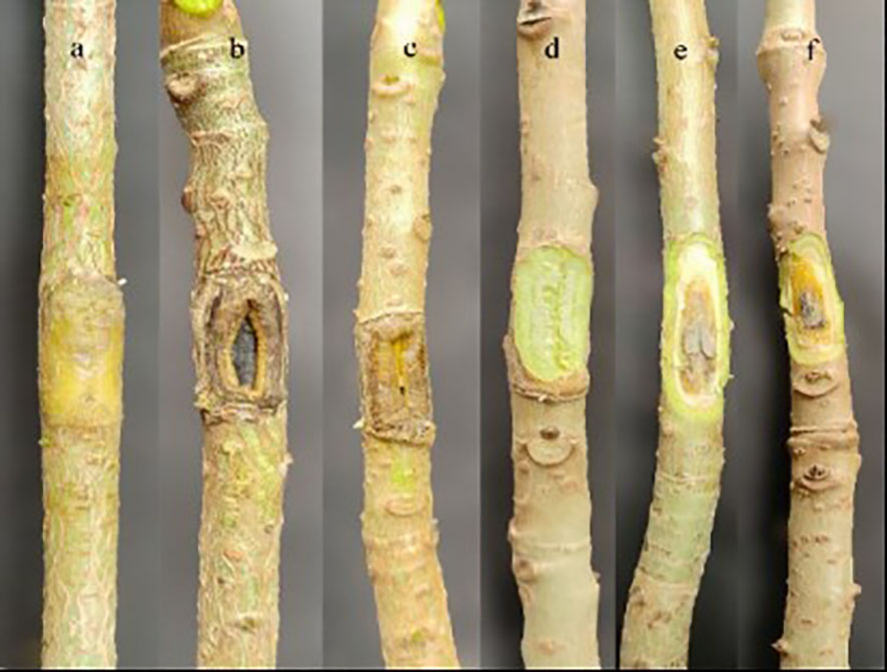
The wound growth condition of 2-years-old *I. polycarpa* after 30 days of inoculation. **(a, d)** represented CK, **(b, e)** represented single inoculations of *B. dothidea*, and **(c, f)** represented simultaneous inoculations of *B. dothidea* and endophytic bacteria B47. After excluding the inoculation length of the mycelial plug, the lesion length of group e was 0.62 ± 0.17, and that of group f was 0.25 ± 0.18.

Wound healing was observed on two-year-old *I. polycarpa* plants with a basal stem diameter of approximately 8 mm at 30 days post-inoculation, as illustrated in **Figure 6**; panels a, b and c depict the healing status, while d, e and f show the tissue conditions following callus scraping. In the CK group, the wounds healed completely, the wounds were rescraped, and no lesions were found. The wounds of the experimental group that were inoculated with *B. dothidea* alone didn’t completely heal, leaving wounds in which the xylem could be observed. The lesions were obvious after the calli were scraped, and the disease index was 36%. The wounds of the *I. polycarpa* plants in the experimental group that was inoculated with endophytic bacteria B47 and *B. dothidea* at the same time had almost completely healed, but the lesions also appeared after re-scratching the wounds. The disease index was 20%, and the relative percentage of endophytic bacteria B47 was 44.44%.

### 3.6 Biological characteristics of endophytic bacteria B47

The physiological and biochemical characteristics of the biocontrol bacterium B47 revealed that the strain did not produce acid in the sugar fermentation test. The catalase reaction was positive, indicating that the biocontrol bacterium B47 contained catalase. The aerobic test and motility test were positive, that was, the biocontrol bacterium B47 was the aerobic bacteria. A positive methyl red test indicated that after the biocontrol bacterium B47 decomposed glucose into pyruvic acid, the pyruvic acid decomposed to produce a large number of acidic substances, causing the pH value to decrease and the methyl red color to change.

**Figure 7:**
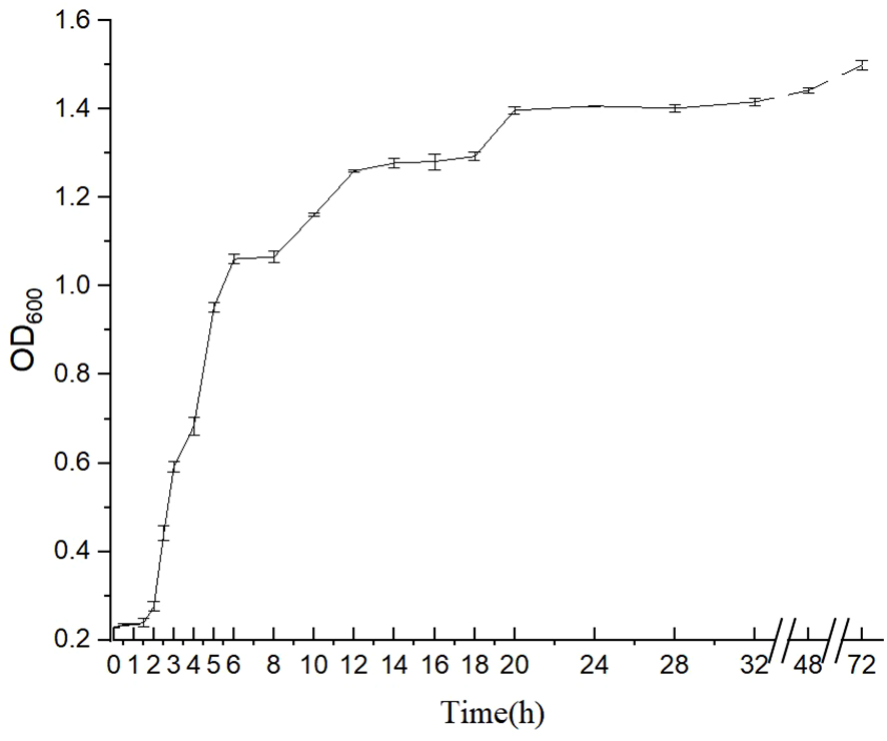
Growth curve of the biocontrol strain B47 in NB medium. OD_600_ refers to the absorbance of a solution measured at a wavelength of 600 nm. This value is positively correlated with the microbial count in the solution, where a higher absorbance indicates a higher microbial concentration.

The growth of biocontrol bacterium B47 in NB medium was in the slow growth period from 0-2 h, the logarithmic growth period from 2-6 h, the slow growth period after 6 h, and the stable period after 20 h (**Figure 7**).

**Figure 8:**
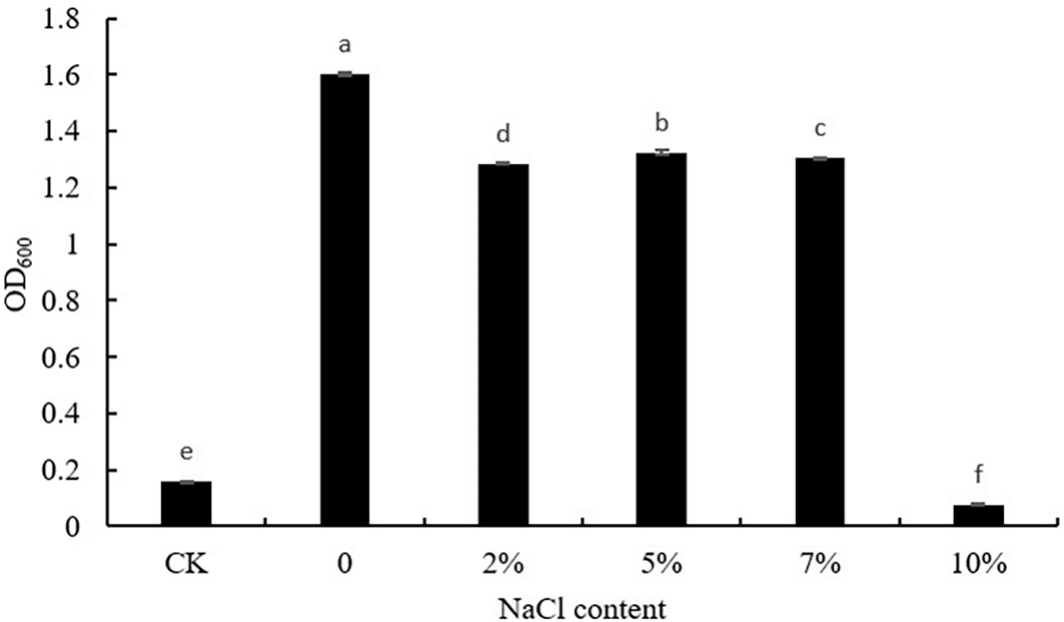
The growth status of biocontrol bacteria B47 under different salt contents. The CK group was blank NB medium, while the NB medium of the experimental group contained endophytic bacteria B47.(*p*<0.01).

In the salt tolerance experiment of biocontrol bacteria B47, it was found that the OD_600_ value of 0% salt content treatment was significantly higher than that of other salt concentration treatments (**Figure 8**) (*p*<0.01). Biocontrol bacteria B47 would be inhibited under different salt concentration contents. The OD_600_ value of the 7% content treatment was significantly higher than that of CK, and the OD_600_ value of the 10% content treatment was significantly lower than that of CK. This indicated that the biocontrol bacteria B47 had a certain degree of salt tolerance and could grow normally at a salt content of 0-7%. When the salt content reached 10%, the salt tolerance ability of the biocontrol bacteria B47 reached the limit and some bacteria died.

**Figure 9:**
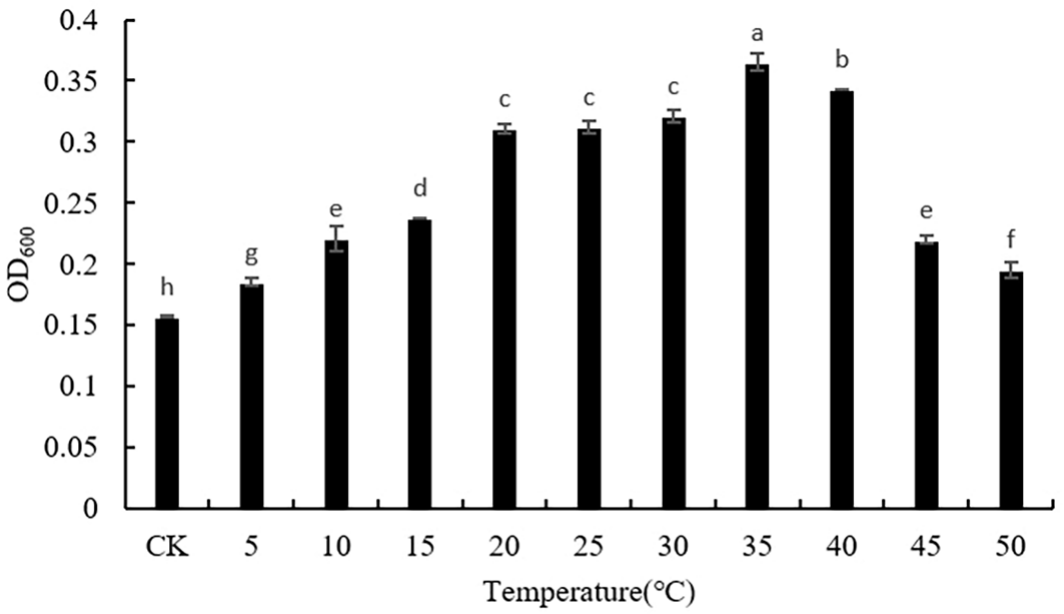
Effects of different temperatures on the growth of biocontrol bacteria B47. The CK group was NB medium without endophytic bacteria B47, and the liquid medium was cooled to room temperature after sterilization. (*p*<0.01).

With increasing temperature, the biocontrol bacteria B47 concentrations first increased but then decreased (**Figure 9**) (*p*<0.01). At 35 °C, the OD_600_ value reached a maximum value, which was significantly greater than those of the other experimental groups, and then decreased with increasing temperature. In the range of 5-50 °C, the OD_600_ values of the test group were significantly greater than those of the CK group, indicating that biocontrol bacteria B47 could grow between 5-50 °C.

**Figure 10:**
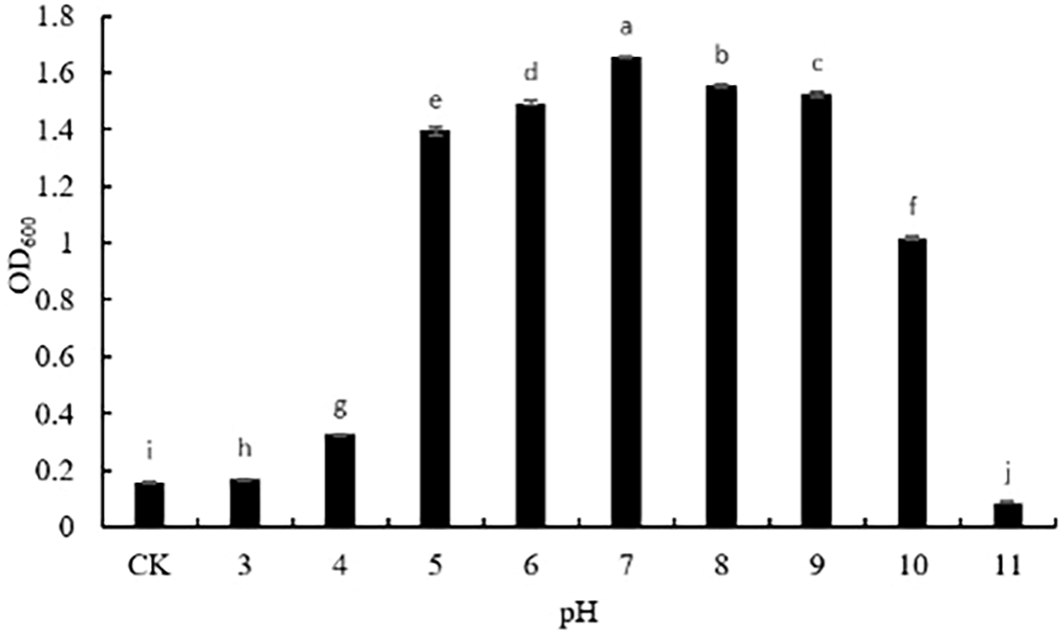
Effects of pH on the growth of biocontrol bacteria B47. The CK group was a blank NB medium with a pH value of 7.2. (*p*<0.01).

In the acid and alkali resistance experiment of biocontrol bacteria B47, except for the treatment with pH 11, the OD_600_ values of the remaining treatments were significantly greater than that of CK (**Figure 10**) (*p*<0.01). This indicated that biocontrol bacteria B47 could survive within the pH range of 3 to 10. The optimal growth pH value was 7, and biocontrol bacteria B47 had strong acid and alkali resistance.

## 4 Discussion

### 4.1 Biocontrol efficacy and potential mechanisms

In the plate confrontation assay, B47 significantly inhibited the mycelial growth of *B. dothidea* without direct contact with the pathogen and caused abnormal colony margin morphology, suggesting that B47 may secrete diffusible antimicrobial substances. Previous studies have shown that bacteria of the genus *Aneurinibacillus* can produce gramicidin S (GS), a diffusible secondary metabolite that is secreted into the extracellular environment and exerts biocontrol effects without requiring direct contact between the producer strain and the pathogen (Berditsch et al., 2007; Alenezi et al., 2022). The non-contact inhibitory phenotype observed in this study suggests that the antifungal activity of *Aneurinibacillus* sp. B47 is unlikely to be mediated by parasitic or competitive niche exclusion mechanisms. This typical antagonistic characteristic is consistent with the core functional traits of the genus *Aneurinibacillus* reported in previous biocontrol studies (Alenezi et al., 2016b).

Previous studies have shown that bacteria of the genus *Aneurinibacillus* can produce antimicrobial peptides such as gramicidin S (GS), an antibiotic that serves as a primary weapon against certain bacteria and fungi (Alenezi et al., 2022). Structurally, GS can insert into the lipid bilayer of fungal cell membranes, disrupt membrane permeability and integrity, cause cytoplasmic leakage, and ultimately lead to the deformation, atrophy, and growth arrest of pathogenic fungal hyphae (Berditsch et al., 2007; Alenezi et al., 2017). At the genetic level, strains of this genus also carry abundant biosynthetic gene clusters for secondary metabolites, including non-ribosomal peptide synthetases (NRPS) and polyketide synthases (PKS), which are responsible for synthesizing various antimicrobial peptides, polyketides, and terpenoids (Alenezi et al., 2016a). These conserved functional gene clusters enable *Aneurinibacillus* strains to produce multiple antagonistic substances simultaneously, forming a composite antifungal system, which explains the stable inhibitory effect of B47 against *B. dothidea in vitro*.

In the biological control of plant diseases, congeneric bacteria have shown promising application prospects. For example, *A. migulanus* strain Nagano reduced the severity of *Dothistroma* needle blight on two-year-old *Pinus contorta* from 5.8% to 1.1%, confirming the biocontrol potential of the genus *Aneurinibacillus* in forest disease management (Alenezi et al., 2016b). *A. migulanus* achieved 50–70% control efficacy against cucumber downy mildew (*Pseudoperonospora cubensis*) in greenhouse trials (Schuster and Schmitt, 2018). Additionally, *Aneurinibacillus aneurinilyticus* OS_25, isolated from holy basil (*Ocimum sanctum*), not only inhibited the growth of *Fusarium oxysporum* but also induced systemic resistance (ISR) in pea plants, effectively reducing the incidence of root rot (Gupta et al., 2022). These findings are highly consistent with the antagonistic activity of *Aneurinibacillus* sp. B47 in the present study and further support the biocontrol potential of the genus *Aneurinibacillus* and its closely related bacteria in plant diseases.

In comparison, *Aneurinibacillus* sp. B47 isolated in this study achieved an *in vitro* inhibition rate of 84.56% against *B. dothidea*, which was at a relatively high level among the reported strains of the genus *Aneurinibacillus*. On detached branches and potted plants, the relative control efficacies of B47 against stem rot were 42.86% and 44.44%, respectively, which was comparable to the field efficacy of the Nagano strain on pine trees (Alenezi et al., 2016a). Although the inhibition rates on detached branches and potted plants were lower than those on culture plates, such differences are commonly observed in biocontrol research (Kashyap et al., 2023). The above phenomenon is likely caused by the following possibilities: (1) the plant internal microenvironment (e.g., nutrients, pH, indigenous microorganisms) affects the colonization and activity expression of *Aneurinibacillus* sp. B47 (Mengistu, 2020); (2) the pathogen infects host tissues in a more complex manner, partially escaping contact with the antimicrobial substances (Han et al., 2016); and (3) the antimicrobial compounds produced by *Aneurinibacillus* sp. B47 may be diluted or degraded within plant tissues (Someya and Akutsu, 2009). To sum up, biocontrol potential varies among different *Aneurinibacillus* strains. Even so, the comparison results indicated that strain B47 was one of the candidates with relatively high activity against *B. dothidea* in this genus.

### 4.2 Biological characteristics and environmental adaptability

The growth and reproduction of biocontrol bacteria are closely related to the environment, and their biocontrol efficacy is often affected by factors such as temperature, humidity, soil and pH. Consequently, the biological characteristics and culture phenotypes of individual strains may differ considerably (Blackburn et al., 2016). Therefore, even strains belonging to the same genus or even the same species can exhibit different biological characteristics. Physiological and biochemical assays of *Aneurinibacillus* sp. B47 in this study showed that the strain is an aerobic, catalase−positive, methyl red−positive bacterium that does not produce acid from sugar fermentation, which differs from some previously reported *Aneurinibacillus* strains. For example, *Aneurinibacillus migulanus* isolated by (Zhang et al., 2016) was positive in the sugar fermentation test, indicating that physiological characteristics vary among strains within the same genus. The growth curve of *Aneurinibacillus* sp. B47 showed that it entered the logarithmic growth phase within 2-6 h, indicating a fast growth rate, which confers distinct advantages for the early prevention, niche competition, and sustainable management of forest diseases.

Environmental adaptability tests showed that *Aneurinibacillus* sp. B47 could grow within the ranges of 5-50 °C, pH 3-10, and 0-7% (w/v) NaCl, with an optimal growth temperature of 35 °C and an optimal pH of 7. *Aneurinibacillus humi* sp. nov., discovered by Kyonggi University, can grow at 20-55 °C, pH 5.0-9.0, and 0-5% (w/v) NaCl (Lee and Lee, 2016). *Aneurinibacillus sediminis* sp. nov., discovered later, can grow at 25-60 °C, pH 6.0-8.8, and 0-2% (w/v) NaCl (Subhash et al., 2017), while *Aneurinibacillus tyrosinisolvens* can grow at 10-30 °C, pH 6.0-6.5, and 0-1% (w/v) NaCl (Tsubouchi et al., 2015). Compared with these closely related strains, *Aneurinibacillus* sp. B47 exhibited a wider pH tolerance range and higher salt tolerance. The strong tolerance of B47 to salt and pH extremes may be related to its isolation from the root environment of *Idesia polycarpa*. This adaptability endows it with excellent viability, broad-spectrum adaptability, and stable colonization ability under diverse soil conditions and climatic regions, rendering it of great application value for green, long-lasting, and broad-spectrum control of forest diseases. Clarifying the biological characteristics and understanding the growth pattern of *Aneurinibacillus* sp. B47 are important bases for evaluating its potential as a biocontrol agent against stem rot of *Idesia polycarpa*.

This study confirmed the biocontrol potential of *Aneurinibacillus* sp. B47 against stem rot of *Idesia polycarpa* Maxim., but many challenges remain. For instance, the complex conditions in the forest, such as variable climate, soil microbial communities, and host physiological status, may affect the colonization ability and biocontrol stability of *Aneurinibacillus* sp. B47. Therefore, further research will focus on elucidating the biocontrol mechanism of *Aneurinibacillus* sp. B47 against dry rot pathogen, exploring its interaction mechanism with *I. polycarpa* and forest microenvironment, and optimizing its stable colonization and efficient disease suppression under field conditions.

## 5 Conclusion

In this study, a total of 179 endophytic strains were isolated and purified from different tissues of healthy *Idesia polycarpa*, including 105 fungi and 74 bacteria. Through the plate confrontation method, an endophytic bacterial strain B47 with an 84.56% inhibition rate antagonistic activity against *Botryosphaeria dothidea*, the pathogen of stem rot of *I. polycarpa*, was screened out, exhibiting an inhibition rate of 84.56%. Based on gene sequence analysis, strain B47 was designated as *Aneurinibacillus* sp. B47.

Detached branch and potted plant assays showed that the relative control efficacies of *Aneurinibacillus* sp. B47 against stem rot of *I. polycarpa* were 42.86% and 44.44%, respectively, and *Aneurinibacillus* sp. B47 was harmless to the host plant, indicating good potential for biocontrol application. Physiological and biochemical characterization revealed that *Aneurinibacillus* sp.B47 is an aerobic bacterium, positive for catalase and methyl red tests, and negative for acid production from sugar fermentation. The strain exhibited broader environmental adaptability, growing at 5-50 °C, pH 3-10, and NaCl concentrations of 0-7%, with an optimal growth temperature of 35 °C, optimal pH of 7, a logarithmic growth phase of 2-6 h, and a rapid growth rate.

In summary, for the first time, an endophytic bacterial strain, *Aneurinibacillus* sp. B47, was isolated from *I. polycarpa*. This strain exhibits an 84.56% inhibition rate against *B. dothidea* and broad environmental adaptability. The biological control effect within *I. polycarpa* was also verified, providing an excellent microbial resource and theoretical basis for the biological control of stem rot.

## Data Availability

The datasets generated and/or analyzed during the current study are available from the corresponding author upon reasonable request.
